# Recent Advances in Structure, Function, and Pharmacology of Class A Lipid GPCRs: Opportunities and Challenges for Drug Discovery

**DOI:** 10.3390/ph15010012

**Published:** 2021-12-22

**Authors:** R. N. V. Krishna Deepak, Ravi Kumar Verma, Yossa Dwi Hartono, Wen Shan Yew, Hao Fan

**Affiliations:** 1 Bioinformatics Institute, A*STAR, 30 Biopolis Street, Matrix #07-01, Singapore 138671, Singapore; ravikumarv@bii.a-star.edu.sg (R.K.V.); yossadh@bii.a-star.edu.sg (Y.D.H.); 2 Synthetic Biology Translational Research Programme, Yong Loo Lin School of Medicine, National University of Singapore, 14 Medical Drive, Singapore 117599, Singapore; wenshanyew@nus.edu.sg; 3 Department of Biochemistry, Yong Loo Lin School of Medicine, National University of Singapore, 8 Medical Drive, Singapore 117597, Singapore

**Keywords:** lipid GPCR, ligand access, orthosteric and allosteric binding sites, drug discovery, antibody, computational methods, prostaglandin receptor, platelet-activating factor receptor, sphingosine-1-phosphate receptor, lysophosphatidic acid receptor, leukotriene receptor, free fatty acid receptor, cannabinoid receptor

## Abstract

Great progress has been made over the past decade in understanding the structural, functional, and pharmacological diversity of lipid GPCRs. From the first determination of the crystal structure of bovine rhodopsin in 2000, much progress has been made in the field of GPCR structural biology. The extraordinary progress in structural biology and pharmacology of GPCRs, coupled with rapid advances in computational approaches to study receptor dynamics and receptor-ligand interactions, has broadened our comprehension of the structural and functional facets of the receptor family members and has helped usher in a modern age of structure-based drug design and development. First, we provide a primer on lipid mediators and lipid GPCRs and their role in physiology and diseases as well as their value as drug targets. Second, we summarize the current advancements in the understanding of structural features of lipid GPCRs, such as the structural variation of their extracellular domains, diversity of their orthosteric and allosteric ligand binding sites, and molecular mechanisms of ligand binding. Third, we close by collating the emerging paradigms and opportunities in targeting lipid GPCRs, including a brief discussion on current strategies, challenges, and the future outlook.

## 1. Introduction

**Figure 1:**
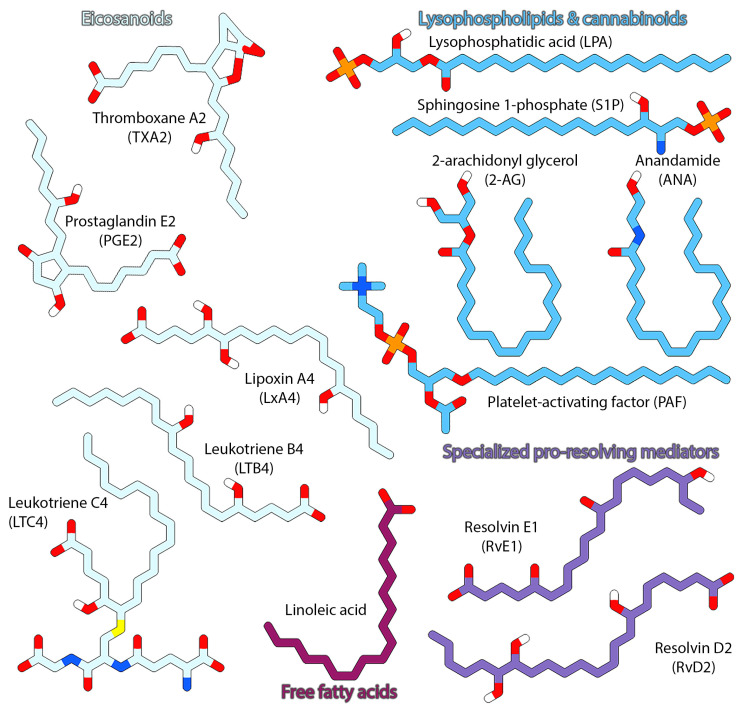
Two-dimensional structures of a few representative examples of endogenous lipid mediators (LMs), belonging to the different classes involved in diverse signaling pathways.

Lipids have attracted the attention of biochemists and cell biologists for several decades, owing to their sheer diversity and for their staggeringly broad cellular and physiological roles. These largely hydrophobic, bioactive molecules modulate a variety of structural and functional aspects central to life, ranging from constituting cellular and organellar membranes, energy storage, protein post-translational modifications, and cell signaling. The role of lipids in cellular signaling processes and their importance for mediating normal cellular and physiological functions have been well recognized [1]. Lipid mediators (LMs) in signal transduction pathways are usually produced locally in response to extracellular stimuli, exported extracellularly, bound to their receptors, and sequestered rapidly, acting as local hormones or autocoids [2]. There are several classes of bioactive lipid mediators, some representative examples of which are illustrated in Figure 1, and their repertoire is continually expanding.

Bioactive lipid mediators can be categorized in three broad classes—(1) arachidonic acid (AA)-derived eicosanoids; (2) lysophospholipids and their derivatives (including endocannabinoids); and (3) omega-3-derived specialized pro-resolving mediators (SPMs). Another important group of lipid mediators comprises fatty acids with medium to long acyl chains. These molecules act as both activators and mediators in several major signal transduction pathways and have critical roles in cell growth, aging and death, intracellular trafficking and cellular migration, inflammation, and immune responses (initiation and resolution). Malfunctional lipid signaling pathways have been found to be at the center of numerous human disorders [3], extensively summarized in Supplementary Table S1. In response to stimuli, lipids exert their roles in signaling pathways by often acting as chemical messengers that bind and activate G protein-coupled receptors (GPCRs) studded on the cell surface. In the present review, we specifically focus on the signaling activities of lipids from the perspective of their cognate G protein-coupled receptors (GPCRs), especially those from the class A (rhodopsin-like) GPCRs and their binding and interactions therewith. We summarize the latest structural, functional, and dynamical insights gleaned from recently elucidated structures and computational studies. We also discuss the implications of these findings for drug discovery and development as well as present new directions and approaches.

## 2. GPCRs Liganded by Lipid Mediators

### 2.1. A Brief Primer on GPCRs

The G protein-coupled receptors (GPCRs) are transmembrane proteins that reside on cell surfaces and govern a plethora of cellular communications and signaling processes. They act as receptors for an extraordinary variety of signaling molecules ranging from light, ions, small organic molecules (odorants, vitamins, neurotransmitters, etc.), hormones (peptide e.g., angiotensin, and non-peptide e.g., estrogen), lipids (sterols, fatty acids, phospho- and sphingolipids and their derivatives), peptides (neuropeptides, complement, etc.), and proteins (glycoproteins, chemokines, etc.) [4]. Once “*activated*”, following the binding of a given signaling molecule or “*agonist*”, the cognate receptor translates the signal intracellularly through a repertoire of heterotrimeric G proteins and other intracellular transducers. In general, GPCRs modulate a wide range of cellular and physiological functions ranging from perception of visual, gustatory, olfactory, and hormonal cues; regulation of mood and behavior; control of internal organ functions; bone development and remodeling; immune system function and inflammatory responses; and maintenance of homeostasis [5,6].

It is understood that endogenous GPCR ligands primarily elicit their responses by binding to a ligand binding pocket located between transmembrane helices, close to the extracellular side, referred to as the orthosteric or canonical site, and is the first step in the GPCR-mediated cell signaling process. The initial ligand binding preferentially stabilizes an active conformation of the receptor characterized by specific conformational changes associated with conserved structural motifs or switches and helical movements [7], which in turn leads to the recruitment of intracellular transducers (G-proteins, β-arrestins, etc.) which relay the signal downstream [8]. The wide-ranging physiological scope and functional diversity of GPCRs is reflected in the sheer number of genes that encode for these receptors in the human genome. GPCRs constitute one of the largest known protein superfamilies, predicted to comprise at least 835 unique members. Based on sequence homology and functional analogy, GPCRs are classified into distinct families or classes, of which classes A, B, C, and F are found in humans [9]. Class A GPCRs are by far the largest group, accounting for ~90% of all receptors, with nearly 50% of them being olfactory receptors [10].

Dysfunction of GPCR signaling activity, either due to inactivation or supra-activation, has been recognized as the causal factor in several human diseases [11,12,13,14,15]. It is no surprise, therefore, that GPCRs constitute an important class of therapeutic targets and are at the center of numerous drug discovery efforts. Sriram and Insel estimated that nearly 35% (~700) of drugs approved for use by the Food and Drug Administration (FDA) and European Medicines Agency (EMA) specifically target GPCRs [16]. Most approved drugs and potential therapeutic candidates in general act as either orthosteric agonists (positive modulators) or antagonists (negative modulators). A small but significant number of molecules also act allosterically by binding to non-canonical sites within the receptors. Approved GPCR-specific drugs are overwhelmingly small molecules (~92%), with nearly 94% of the approved drugs and 85% of molecules in clinical trials targeting class A receptors. Undoubtedly, factors such as the role GPCRs in diverse vital physiological processes, the accessibility of the ligand-binding orthosteric site at the cell surface, and the existence of potential druggable allosteric sites contributed immensely to the therapeutic utility of targeting GPCRs, both in academia and industry. It is pertinent, therefore, to note that among the potential druggable GPCRs, just over a quarter (~107) are currently targeted by approved drugs, while potential drug candidates have reached clinical trials for another 67 receptors. More than half of the total number of potentially druggable GPCRs (~221) are brimming with untapped potential and are ripe for exploration, including a large number of GPCRs liganded by lipid or lipid-like molecules [10,17,18].

### 2.2. Lipid GPCRs and Associated Drugs

**Figure 2:**
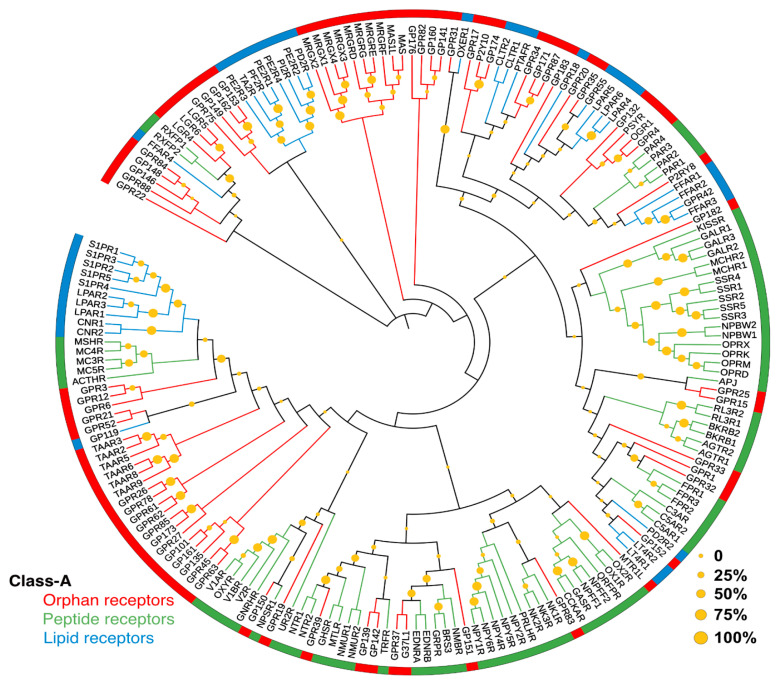
Phylogenetic tree of 193 Class-A lipid, peptide, and orphan receptors. The alignment of the sequence of the transmembrane region was downloaded from the GPCRdb [17] database and the phylogenetic tree was constructed using the maximum likelihood tree method using 1000 replicates in MEGAX software suite [20]. The circular graphical representations of phylogenetic trees were displayed in a circular tree layout using iTOL version 5 [21]. The lipid, peptide, and orphan receptors are shown in blue, green, and red colors, respectively. Bootstrap confidence values for the nodes are given by yellow spheres.

As many as 50 different GPCRs are estimated to be liganded by lipid mediators, of which 36 unique receptors belong to class A (rhodopsin-like) GPCRs [19]. These include several “orphan” receptors, for which cognate ligands are yet undetermined. Phylogenetically, lipid receptors are also closer to several other receptors liganded by peptides/peptidic molecules, some of which are promiscuously activated by both lipids and peptides (e.g., FPR2/ALX). The phylogenetic relationships among the lipid, peptide, and orphan receptors are illustrated in Figure 2.

As evident from Supplementary Table S1, LMs are involved in numerous and diverse physiological processes, and the dysregulation of lipid receptors is known to be associated with several human disease conditions and disorders including tumorigenesis, cancer cell proliferation, and metastasis [19]. The recent advances in the structural biology of lipid GPCRs have significantly contributed to our understanding of these receptors and greatly increased pharmacological interest in them. Lipid GPCRs, thus, represent ideal targets for drug design, discovery, and development efforts, which is reflected in the number of drugs molecules approved for use or under trials in the last five years. Since the compilation of the curated estimate of Sriram and Insel [16] (November 2017), as many as 41 new GPCR-targeting drugs have been approved for use by FDA/EMA (as of September 2021). In the same period, an additional seven new GPCR-specific drugs were approved by the Japanese Pharmaceuticals and Medical Devices Agency (PMDA) [19]. Of these newly approved drugs, those targeting lipid GPCRs are: cannabidiol (CB_1,2_), siponimod fumarate (S1P_1,5_), ozanimod hydrochloride (S1P_1,5_), and ponesimod (S1P_1_). In the same period, treprostinil monosodium salt (IP) was approved by EMA in 2020, having been FDA-approved in 2001. Omidenepag isopropyl (EP_2_) was approved by PMDA in 2018. The updated list of approved drugs targeting lipid GPCRs can be found in Supplementary Table S1.

Nearly all the approved lipid GPCR drugs are involved in neuroactive ligand-receptor interaction pathway (all pathways mentioned are KEGG pathways [19]). The prostanoid drugs target vascular smooth muscle contraction and platelet activation pathways, are used to treat vascular diseases, ulcers, allergies, glaucoma, and ocular hypertension and are used as oxytocics, luteolytics, vasodilators, anti-secretories, and anti-inflammatories. Leukotriene drugs are used to treat asthma, allergic rhinitis, and epilepsy. PAF drugs target inflammatory mediator regulation of the TRP channels pathway, and the only approved drug targeting PAF is rupatadine (approved by PMDA in 2017), used in the treatment of allergic rhinitis. S1P drugs target the sphingolipid signaling pathway. are used to treat multiple sclerosis, ulcerative colitis, and lupus. and are used as anti-ulceratives and immunosuppressants. Cannabinoid drugs are used to treat obesity and are used as anti-emetics, anti-convulsant, anti-epileptic, and analgesics. Notably, the cannabinoid Δ^9^-tetrahydrocannabinol (THC) has been approved by FDA in 1985 (synonyms: nabilone/dronabinol), and the related cannabinoid cannabidiol (CBD) has been recently approved by FDA in 2018 to treat epilepsy and as an analgesic. The only free fatty acid (FFA) drugs in this dataset are rosiglitazone (FDA approval in 1999) to maintain glycemic control in Type 2 diabetes, and icosapent (FDA approval in 2012) used as a nutraceutical; both target FFA_1_. Notably, there are no known approved drugs targeting LPA receptors, FPR_2_, and CMKLR_1_.

Mizuno and Kihara have compiled a comprehensive list on the lipid GPCR drugs in clinical trials [22]. Most are intended for largely similar indications as those of already approved drugs. For example, the investigational prostanoid drugs have been trialed for vascular diseases, asthma, and others, much like the existing approved drugs. Some existing approved drugs are being trialed for different indications, though usually with common underlying physiological pathways, such as alprostadil, which was approved for erectile disfunction and is trialed for cardiovascular diseases. One example of a differing indication is cannabidivarin, a close analogue of approved cannabidiol differing by just a two-carbon length, trialed for autism spectrum disorder and Prader-Willi syndrome, while cannabidiol is approved to treat epilepsy and pain.

## 3. Progress in Structural Biology of Lipid GPCRs

For the longest time, obtaining diffraction quality crystals for structure determination of GPCRs, or membrane proteins in general, proved to be a great obstacle towards understanding their structural biology. However, the past two decades have seen dramatic progress in this area owing to the improvements and development of new tools, techniques, and novel approaches in the protein engineering, expression systems, purification, and crystallization of receptors. These include disulfide bridge engineering, lipidic cubic phase, T4-lysozyme/BRIL fusion, receptor stabilizing antagonist tool compounds and antibodies, and X-ray microdiffraction [23,24,25]. The recent emergence of cryo-electron microscopy (cryo-EM) methods has further pushed the envelope in providing structural insights into GPCR structural biology [26,27]. 

We shall briefly mention some caveats related to the experimental techniques and conditions to resolve the structures. In X-ray crystallography, the crystal lattice is needed for diffraction, and the crystal packing might result in artifactual conformations of the involved residues. In the context of lipid GPCRs, this may constrain the conformation of extracellular loops. Some resolved cholesterol molecules may also be crystal packing artifacts [28]. In the context of oligomerization, an observation of GPCR oligomers in an X-ray crystal structure may be only due to crystal packing [29]. Buffer compositions, detergents, additives, and other conditions favorable for crystallization and cryomicroscopy may not be congruent with the native physiological conditions [29]. For example, fusion protein or antibody is often introduced to stabilize ICL3 in crystal structures. Several point mutations may also be introduced for thermodynamic stability to form crystal lattice, although they are typically far from the crucial sites and the mutant construct is ensured to still be functional. In cryo-electron microscopy (cryo-EM), the abovementioned artifacts related to crystal lattice are absent, but a different set of challenges is present, such as anisotropy and the tricky interpretation of resolution of a cryo-EM structure [30]. Computational techniques such as MD simulations may help resolve some of these artifacts [28].

Due to the rapid and sustained progress in GPCR structural biology, currently there are as many as 574 structures from 105 unique GPCRs in the Protein Data Bank, including 41 structures covering 17 unique lipid GPCRs, mostly from humans [17,18]. Currently, receptors for several established classes of lipid mediators, including lysophospholipids (S1P and LPA), prostanoids (PGD_2_ and PGE_2_), leukotrienes (LTB_4_ and LTD_4_), free fatty acids, platelet-activating factors, and cannabinoids, are available. These receptors are solved in the presence of agonists, antagonists, or allosteric modulators, with some of them co-crystalized and solved in the presence of bound G proteins. The different lipid GPCRs with solved structures from humans and other organisms are summarized in Table 1.

Overall, lipid GPCRs share the highly-conserved seven transmembrane helix (7TM) fold characteristic of GPCRs, and the structural and functional aspects of the architecture have been reviewed in detail elsewhere [5,6,59]. Upon closer examination, the lipid receptor structures revealed subtle, as well as pronounced, variations among themselves and with respect to other class A GPCRs. In the subsequent sections, we discuss in detail these variations and how they impact receptor function, by reviewing the new and emerging structural, functional, and dynamical insights into lipid GPCRs, illuminated from crystallographic, biophysical, mutational, and computational studies.

### The First Lipid GPCR Structure

**Figure 3:**
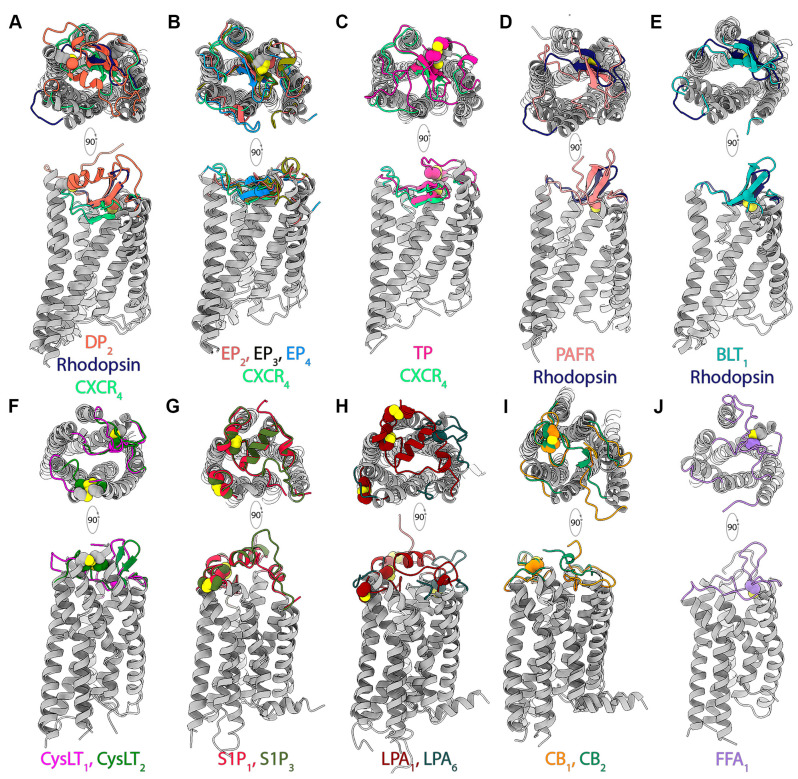
Organization of the extracellular domain in representative lipid receptor structures belonging to (**A**) DP_2_ (dark orange), (**B**) EP_2,3,4_ (red, olive, blue, respectively), (**C**) TXA_2_ (dark pink), (**D**) PAFR (coral), (**E**) BLT_1_ (cyan), (**F**) cysLT_1,2_ (magenta, dark green, respectively), (**G**) S1P_1,3_ (crimson, olive green, respectively), (**H**) LPA_1,6_ (maroon, slate, respectively), (**I**) CB_1,2_ (orange, sea green), and (**J**) FFA_1_ (purple). The structures of rhodopsin (navy blue; PDB ID: 4ZWJ) and CXCR4 receptors (spring green; PDB ID: 3ODU) are superposed with the lipid receptors, where appropriate, to indicate the relative positions of ECL2. The cysteine residues from the lipid receptors involved in disulfide bridges are highlighted using sphere representation.

In 2012, Hanson et al. reported the first structure of a lipid GPCR, that of the human sphingosine 1-phosphate receptor subtype 1 (S1P_1_) complexed with a sphingolipid mimic antagonist, which revealed several novel structural and functional features hitherto unobserved in the earlier GPCR structures [40]. The most distinguishing feature concerned the architecture of the extracellular domain. In the S1P_1_ structure, the N-terminus was organized into a short helical segment (referred to as the N-helix below), which packed against the three extracellular loops (ECL1, 2 and 3). Another novel feature was the presence of an intra-loop disulfide bond in ECL2 and ECL3, while the TM3-ECL2 disulfide bond observed in several other class GPCRs was absent. Such a novel organization of the extracellular domain was previously unrecognized, and it appeared to render the orthosteric site inaccessible from the extracellular milieu (Figure 3G). Relative to other GPCRs of known structure S1P_1_, a pronounced gap was exhibited between TM1 and TM7, facilitated by the repositioning of TM1 and TM2, which the authors reasoned could serve as the port of entry for the amphiphilic antagonist (ML056) and agonist (S1P). The structure also revealed a highly amphipathic orthosteric site, illustrative of the zwitterionic-hydrophobic nature of S1P receptor agonists and antagonists. In the structure, the phosphonate and amine moieties of the antagonist ML056 favorably interacted with a charged region of the binding pocket comprising R120^3.28^ and E121^3.29^, respectively, while its acyl tail is buried in a hydrophobic sub-pocket formed by aliphatic and aromatic residues from TMs 3, 5, 6, and 7. The structural observations were consistent with previously reported mutagenesis studies [60], while the structure-activity relationship and docking studies revealed how acyl tail length and phenyl ring substitution patterns of ligands determined antagonism or agonism. Another interesting observation from the structure relates to residue R292^7.35^, located on the extracellular end of TM7, whose cationic sidechain is projected away from the 7TM core. Mutation of R292^7.35^ has been shown to render the receptor non-responsive to S1P, and structural modeling studies predicted that the residue formed a salt-bridge interaction with the phosphate group of S1P [60]. Although, no such interaction was observed in the crystal structure, based on earlier mutational studies and the present structure the authors speculated that R292^7.35^ acted as a “cationic lure”, projecting its side chain into the hydrophobic milieu of the membrane upper leaflet to attract phospholipid.

The structure of S1P_1_ brought forth several illuminating insights into the how lipid binding GPCRs potentially interact with the solvent and membrane milieus, how they potentially recognize and capture cognate ligands, and how the seemingly pliable nature of their orthosteric sites affects agonism and antagonism. Since the publication of the S1P_1_-ML056 structures, several more structures of lipid GPCRs liganded by diverse lipid mediators, complexed with agonists, antagonists (orthosteric and allosteric), G-proteins, antibodies/nanobodies, etc., have been reported in the past decade. These structures have provided unprecedented insights into the structural diversity and functional mechanisms of lipid GPCRs, which we will elaborate in the subsequent sections. In the subsequent sections, we will highlight the distinguishing features of lipid receptors, both subtle and pronounced, and how they affect the lipid mediated agonism and antagonism of their cognate receptors.

## 4. Structural and Functional Features of Lipid GPCRs

### 4.1. Organization of Extracellular Domains in Lipid GPCRs

The structural diversity of ECL2 configurations, and their role in mediating ligand binding, are well recognized in GPCRs [61]. Currently, structures of lipid receptors liganded by lysophospholipids (S1P_1_, S1P_3_, LPA_1_ and LPA_6_), prostaglandins (DP_2_, EP_2_, EP_3_ and EP_4_), leukotrienes (BLT_1_), cysteinyl leukotrienes (cysLT_1_ and cysLT_2_), thromboxanes (TP), free fatty acids (FFA_1_), platelet-activating factors (PAFR), and cannabinoids (CB_1_ and CB_2_) are available (Table 1). A marked feature of these lipid receptor structures is the inherent diversity in organization of the extracellular domain and its implications for ligand access. In the currently available structures of lipid GPCRs, the extracellular domain appears to adopt one of four distinct configurations characterized by how the N-terminus and extracellular loop 2 (ECL2) are organized (Figure 3). In each lipid receptor, the N-terminus and ECL2 are either organized into a short helical segment and a β-hairpin, respectively, or remain unstructured or exist in a combination thereof. The organization of the N-termini and ECL2 segments into secondary structural elements appear to be independent of each other. The ECL2 is organized into a β-hairpin in the prostaglandin D2 (DP_2_) and E2 (EP_2_, EP_3_ and EP_4_) receptors, the thromboxane A2 receptor (TP), the leukotriene receptor (BLT_1_), the cysteinyl leukotriene receptors (cysLT_1_ and cysLT_2_), and the platelet-activating factor receptor (PAFR), while those in the lysophospholipid receptors (S1P_1_, S1P_3_, LPA_1_ and LPA_6_), the free fatty acid receptor (FFA_1_ or GRP40), and the cannabinoid receptors (CB_1_ and CB_2_) have an unstructured coil-like ECL2 (Figure 3). It must be noted that among the four available structures of cysLT_2_ receptors, a clearly structured ECL2 is observed only in one structure (PDB ID: 6RZ8), suggesting that the conformation of the ECL2 is malleable and not fixed, at least in some receptors. In receptors with the β-hairpin ECL2, only the DP_2_ receptor has the N-terminus organized into a helical segment (N-helix). In contrast, the S1P, LPA, and CB receptors possess an N-helix coupled to unstructured ECL2 segments. Only the FFA_1_ receptor lacks both a structured ECL2 segment and N-terminus (Figure 3J). Further, those receptors with the β-hairpin ECL2 exhibit differences among themselves with respect to how the ECL2 is placed relative to the canonical orthosteric site. In case of DP_2_, PAF, and BLT_1_ receptors, the ECL2 β-hairpin is only partly buried within the orthosteric pocket, resembling the arrangement observed in the CXCR4 receptor [62]. In contrast, the ECL2 β-hairpin in the TP, EP_2_, EP_3_, and EP_4_ receptors is buried deeper within the orthosteric pocket such as that in rhodopsin [63]. Although there seem to be clear distinctions among the lipid receptors with respect to the organization of the extracellular domains, the differences exhibited within a given set of receptor structures (e.g., cysLT_1,2_, CB_1,2_) must be looked at with utmost care. Factors such as crystal packing, crystallization conditions, and structure resolutions must be taken into consideration before assigning functional significance to the variations in these structural elements.

Another interesting observation relates to the disulfide bonds present in the extracellular domain. The disulfide bonds of the extracellular regions are suggested to confer structural stability to the receptors, and the TM3 (C^3.25^)-ECL2 disulfide bond is highly conserved across several class A GPCRs [64]. The TM3-ECL2 disulfide bond is also present in most of the lipid GPCRs of known structure. However, this conserved interaction is lacking in the human lysophospholipid (S1P_1_, S1P_3_, and LPA_1_) and cannabinoid (CB_1_ and CB_2_) receptors. Apart from the TM3-ECL2 disulfide bond, some lipid receptors also possess additional disulfide bonds and are summarized in Supplementary Table S2 and illustrated in Figure 3.

Lipid receptors exhibit diverse organizational styles with respect to the N-terminus (helical or unstructured), ECL1, ECL2 (β-hairpin or unstructured), and ECL3, and the interactions among these elements (via non-covalent interactions or disulfide bonds). In several lipid GPCRs, the extracellular domain packing governed by ECL2 and the N-termini has been suggested to play a critical role in governing orthosteric site access from the extracellular milieu and dictate how lipid ligands gain access to the pocket. Structural evidence suggests that the canonical orthosteric site is completely occluded from the extracellular milieu in S1P_1,3_, DP_2_, EP_3,4_, TP, PAFR, FFA_1_, and CB_1,2_ receptors, while the pocket is partially accessible via solvent accessible channels in cpBLT_1_ (from guinea pig; PDB ID: 5X33) and EP_2_ (PDB ID: 7CX2). In contrast, the ligand binding pocket is fully accessible from the extracellular milieu in case of LPA_1_, LPA_6_, and hsBLT_1_ (from human; PDB ID: 7K15). The role these extracellular domain elements play in modulating ligand recognition and binding as deduced from crystallographic, experimental, and computational studies is highlighted in the following sections.

### 4.2. Mode and Dynamics of Lipid Ligand Access

Historically, it had been understood that GPCR ligands primarily accessed the orthosteric site of the receptor, by partitioning out of bulk solvent directly from the extracellular milieu. However, the issue of ligand access in case of lipid receptors is not straightforward and is more nuanced, primarily owing to two broad reasons—(1) the amphipathic/lipophilic nature of their ligands and (2) the complete or partial occlusion of the orthosteric site from the extracellular milieu. Moreover, many lipid mediators such as prostanoids, leukotrienes, and endocannabinoids are synthesized de novo from membrane phospholipids and act locally on neighboring cells [65]. In addition, for other lipid ligands that require transcellular transportation there exist specialized mechanisms to deliver them to their site of action. For example, one of the ways in which transcellular transport of S1P is achieved is through complexation with high-density lipoproteins (HDL), which in turn delivers S1P to its cognate receptor in a process involving physical interaction between the HDL and S1P receptors located on the plasma membrane [66]. Similarly, agonist LPA molecules exist in complex with their biosynthetic enzyme, the phospholipase autotaxin (ATX) on the surface of exosomes, the site of their synthesis. Such an LPA-loaded ATX exosomal carrier system has been demonstrated to activate LPA receptor signaling via fusion with the target cell membrane [67]. It has, therefore, long been believed that, unlike hydrophilic diffusible ligands, many of the lipid ligands access the ligand binding pocket by first partitioning into the lipid bilayer followed by lateral diffusion/translation along the membrane plane.

Strong evidence to support the idea of hydrophobic ligands entering and exiting ligand binding pockets in GPCRs from within the membrane came from comprehensive crystallographic, biochemical, and computational studies on rhodopsin, the prototypical class A GPCR [68,69,70,71,72]. Building upon this nearly a decade’s worth of work, Heck and co-workers, using skeleton search algorithms and molecular docking, identified a contiguous channel, inclusive of the orthosteric site with a potential 11-*cis*-retinal ingress site (between TM1 and TM7) and an all-*trans*-retinal egress site (between TM5 and TM6) within the membrane-embedded regions [73]. Studies have suggested that such a translocation pathway is putatively conserved in class A GPCRs and has been shown to be used for accessing the orthosteric binding pockets in the opsin [74] and adenosine A_2A_ receptor (A_2A_R) [75] by noncognate ligands such as lipophilic detergent molecules and membrane cholesterol, respectively. Based on homology models of the human CB_2_ receptor, Pei et al. designed isothiocyanate covalent labelling experiments and suggested that the cannabinoid AM841 enters the orthosteric site from within the lipid bilayer [76], while Hurst et al. proposed that the entry port for the endocannabinoid 2-arachidonoylglycerol (2-AG) could potentially be located between TM6 and TM7 [77].

The most compelling structural evidence for the “lipid binding via membrane” model was offered by the structure of the first lipid GPCR, the human S1P_1_ receptor. The S1P_1_ structure revealed several novel features including (1) a hitherto unseen extracellular domain architecture that occluded the orthosteric site from the extracellular milieu, (2) the presence of a potential opening between TM1 and TM7 that could serve as the entry port for lipidic ligands, and (3) presence of a functional important cationic residue (R292^7.35^) at the extracellular end of TM7 that could act as a “cationic lure” for attracting lipid ligands containing anionic headgroups from within the membrane [40]. Based on the S1P_1_ structure, mutagenesis data, and receptor saturation binding experiments, the authors—(1) concluded that the tight packing of the N-helix with ECL1 and ECL2 occluded the orthosteric site from the extracellular milieu and, therefore, (2) proposed that S1P enters the pocket possibly via the opening between TM1 and TM7 from within the membrane. In the last decade since the publication of the S1P_1_ receptor structure, as elaborated in Section 3, receptor structures for several other classes of lipidic ligands have been determined. In several of these receptors, the orthosteric site is either fully or partially occluded from the extracellular milieu due to the architecture of the extracellular domain (Section 4.1). Similar to S1P_1_, structures of several of the other lipid receptors including S1P_3_, DP_2_, TP, CB_1_, and CB_2_ show a significant gap between TM1 and TM7 that can been accessed by ligands to enter the orthosteric pocket via the membrane [51,52], while the antagonist-bound EP_4_ receptor structures (PDB IDs: 5YWY, 5YHL) show the presence of lipidic detergent molecules bound in the cleft between TM1 and TM7 [36].

Computational simulation studies have further helped in delineating the atomistic details of the mode of ligand access in S1P_1_. McCammon and co-workers, while studying S1P_1_ dynamics using molecular dynamics (MD) simulations observed that the TM1-TM7 gap increased during simulation, while also reporting on the interaction of bilayer palmitoyl-oleoyl-phosphatidylcholine (POPC) lipid molecules with the TM1-TM7 cleft in 11 out of 12 independent simulations [78]. Stanley et al. further provided a clearer breakdown of the molecular events leading up to diffusion of ML056 into the membrane milieu and subsequent entry into S1P_1′_s orthosteric pocket using ~800 μs of unbiased MD simulations [79]. Partial unbinding of bound agonist, widening of the TM1-TM7 gap, and the entry of a bilayer POPC molecule through the gap towards the orthosteric site occurred in equilibrium MD simulations of DP_2_ in a POPC bilayer. Analyses of the MD trajectories revealed the role of a “cationic tetrad” of residues located at the TM1-TM7 entry port in capturing anionic ligands from the membrane. Mutation of these cationic residues reduced receptor-signaling activity in in vitro functional assays [32]. Although not occupying structurally equivalent positions, functionally, R284^7.32^ of the cationic tetrad in DP_2_ appears to play a similar role as R292^7.35^ in S1P1, that of a “cationic lure” to attract anionic headgroup of lipid molecules from the lipid bilayer. A cursory examination reveals that the presence of one or more cationic residues at the TM1-TM7 entry port is observed in other lipid receptors such as LPA_1_ (K294^7.36^), *dr*LPA_6_ (R281^7.32^, K26^1.31^), EP_2_ (R302^7.40^), EP_3_ (R333^7.40^), EP_4_ (R316^7.40^), TP (R295^7.40^), CB_1_ (K373^7.29^, K376^7.32^), and CB_2_ (K278^7.32^, K279^7.33^), and in some cases has been observed to directly interact with anionic groups of the bound ligands. These observations suggest that these lipid receptors may use common mechanisms for the capture and binding of ligands.

Structural data also show that there are cases where the port of entry for lipidic ligands is not obvious or they use a different entry port. For example, the agonist-bound EP_3_ [PDB ID:6AK3] shows TM1 and TM7 packed tightly together with the orthosteric site inaccessible from both the extracellular side and membrane [34]. Similarly, the cysLT_1_ and cysLT_2_ receptors have a disulfide bridge (C14^1.23^-C267^7.25^ in cysLT_1_, C31^1.25^-C279^7.27^ in cysLT_2_) tethering the extracellular ends of TM1 and TM7 together, precluding ligand entry via the TM1-TM7 gap. Antagonist-bound crystal structures of cysLT receptors suggest that a potential entry port could exist between TM4 and TM5 (PDB IDs: 6RZ4, 6RZ5). Similarly, the TM1-TM7 gap is narrower in the structures of human LPA_1_ (PDB IDs: 4Z34, 4Z35, 4Z36) and zebrafish LPA_6_ (PDB ID: 5XSZ) compared to S1P_1,3_, suggesting that the ligand entry port is not located at the TM1-TM7 interface in these receptors. Instead, the ligands may access the orthosteric pocket via a hydrophobic cleft between TM4 and TM5 observed in the zebrafish LPA_6_. It is also suggested that, despite sharing significant sequence and structural similarity with S1P_1_ (41% sequence identity in TM region), based on critical residue substitutions that led to significantly smaller and less variable openings between TM1 and TM7 compared to S1P_1_ during comparative molecular dynamics (MD) simulations [42], LPA_1_ ligands may access the binding pocket directly from the extracellular side and not via the membrane. The structural features of lipid receptors presented in this section deepen our understanding about lipid ligand recognition and selectivity mechanisms. It is amply evident that despite similarities in the nature of their ligands, among lipid GPCRs there exist multiple modes of ligand access. Ligands may preferentially access the binding pocket via the membrane through entry ports at TM1-TM7 (S1P_1,3_, DP_2_, TP, CB_1,2_, EP_2,3,4_) or TM4-TM5 (CysLT_1,2_, *dr*LPA_6_) or directly from the extracellular milieu (LPA_1_). However, it should be noted that the picture of how lipid receptors initially interact and bind their ligands is incomplete. It is likely that certain receptors may exhibit the ability to capture ligands via the membrane and directly from the extracellular milieu or through modes hitherto unrecognized. Although not a GPCR, the bacterial lysophospholipid transporter (LplT) has been shown to bind its cognate ligands both via the membrane and from the extracellular milieu [80], and certain lipid GPCRs may exhibit similar features depending on the ligand. 

### 4.3. Canonical and Non-Canonical Ligand Binding in Lipid Receptors

The canonical ligand binding site in GPCRs is referred to as the orthosteric site and is principally located in the region between ECL2 on the extracellular side and the highly conserved residue position 6.48 (mostly occupied by Trp) towards the intracellular side. Residues from TMs 3, 5, 6, and 7 predominantly constitute the binding pocket along with some residues from the ECLs (mainly ECL2) and the N-terminus in certain cases. In this section, we discuss the features and modes of ligand binding within the orthosteric sites of lipid GPCRs, highlighting commonalities and differences, as revealed from recent studies.

Most lipid ligands of GPCRs are hydrophobic or amphipathic molecules, and it is, therefore, not surprising that the orthosteric sites in lipid GPCRs are also largely hydrophobic or amphipathic. As inferred from Figure 1, physiological lipid mediators such as sphingolipids (S1P), glycerophospholipids (LPAs, PAFs), and fatty acyls (prostaglandins, thromboxanes, leukotrienes, free fatty acids, endocannabinoids, and lipoxins), among others, share common chemical and structural features while exhibiting variations in nature of polar groups and/or headgroups and acyl chain length. Aromatic moieties are also an oft-observed feature in lipidic ligands, especially in synthetic small molecule drugs and drug candidates. The characteristics of the orthosteric ligand binding pockets in lipid binding GPCRs, in terms of residue composition and their distribution, are indicative of the type of ligands a given receptor shows preference for. Depending on the cognate lipid ligand, the receptors have complementary polar/charged residues that interact with polar/charged moieties in the ligands, while their aliphatic and aromatic moieties occupy sub-pockets populated by aliphatic and aromatic residues. Understanding the nature and mode of ligand binding holds tremendous importance for rational design of specific (receptor-type and subtype) agonists and antagonists.

**Figure 4:**
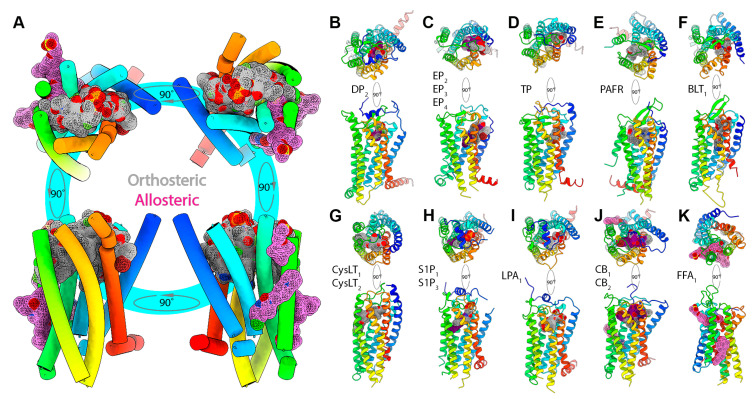
(**A**) Distribution of bound ligands in the orthosteric site (surface mesh representation with carbon, oxygen, nitrogen, and phosphorus atoms colored grey, red, blue, and orange, respectively) and allosteric sites (pink surface mesh) in a representative lipid receptor structure illustrated using cylinder (helices only) representation. Distribution of bound orthosteric agonists (surface mesh representation with carbon, oxygen, nitrogen, and phosphorus atoms colored purple, red, blue, and orange, respectively) and antagonists (grey surface mesh), and allosteric antagonists (pink surface mesh) bound to structures belonging to (**B**) DP_2_, (**C**) EP_2,3,4_, (**D**) TXA_2_, (**E**) PAFR, (**F**) BLT_1_, (**G**) cysLT_1,2_, (**H**) S1P_1,3_, (**I**) LPA_1_, (**J**) CB_1,2_, and (**K**) FFA_1_.

Figure 4A shows the distribution of all lipid ligands (summarized in Table 1) within the orthosteric pocket of a representative lipid GPCR. Within the large contiguous canonical site, stretching between the TM1-TM7 and TM4-TM5 interfaces, ligands occupy different sub-regions depending on the nature of the ligand and the type of receptor. Based on the binding mode, different ligands interact with various residues from the extracellular halves of the TM helices as well as the extracellular domain elements (Figure 4A). Most ligands co-crystallized with their cognate lipid receptor bind centrally and largely occupy a similar region within the orthosteric site. The binding modes of ligands in the DP_2_ (Figure 4B) and EP_2,3,4_ (Figure 4C) receptors offer a compelling demonstration for the plasticity of the ligand binding pocket in lipid receptors. The receptors EP_2-3_ and DP_2_ are activated by the prostaglandins PGE_2_ and PGD_2_, respectively. Both PGE_2_ and PGD_2_ are structurally similar, and possess two aliphatic chains, the α-chain ending in a carboxyl group (acidic tail) and the ω-chain, joined together by a central hydroxycyclopentanone moiety, the E-ring (Figure 1). In case of DP_2_, the ligands bind centrally, with ligands adopting a U-like conformation, engaging the N-helix and ECL2 with their carboxylate groups pointing inwards. In contrast, PGE2 molecules in EP_2–4_ receptors show ligands adopt a more linear conformation, packing against TM1 and TM7 with their acyl tails pointing towards W^6.48^, assuming the opposite orientation as that of PGD2. In the EP_2–4_ receptors, the bound PGE_2_ molecule largely adopts an L-shaped conformation near the confluence of TM1-TM2-TM7 with the α-chain, E-ring, and ω-chain occupying distinct sub-pockets within the orthosteric site. The carboxyl group of the α-chain points towards the membrane-extracellular milieu interface and interacts with polar/charged residues from ECL2, TM2, and TM7 (R^7.40^), while its aliphatic portion is surrounded by conserved hydrophobic and aromatic residues. The ω-chain on the other hand is buried within a hydrophobic sub-pocket formed by residues from ECL2, TM3, and TM7. The E-ring packs against TM1 and TM2, with its hydroxyl and carbonyl groups forming hydrogen bonds. Ligands bound to the TP/TXA_2_ receptor (Figure 4D) binds similar to PGE2 in EP_2–4_, with a part of the ligand wedged between TM1 and TM7.

In case of the S1P_1_ (inactive) and S1P_3_ (active) structures, the linear antagonist and agonist molecules adopt extended conformations, with the antagonist ML056 showing a slight bend, while bound to the long penetrating tunnel-like binding pocket. While the tunnel structure is shallower in antagonist-bound S1P_1_, it is longer in the agonist S1P-bound S1P_3_ with the longer acyl tail of S1P extending further through a gap between TM4 and TM5 (Figure 4H). S1P_1_ mutagenesis, molecular modeling, and docking, as well as structure-activity relationships studies, have shown that increments in acyl chain length and switching phenyl ring substitution patterns could convert antagonists to full agonists. Further, the authors elaborate how S1P_1_ agonism is achieved by known agonists possessing a polar headgroup (class I) and other small molecules lacking a polar headgroup (class II) [40,60]. Molecular simulations have helped further explain how differences in acyl length affects activation [81]. In S1P_3_, with the help of assays to evaluate receptor signaling (cAMP accumulation and TGFα secretion), it was established that length of acyl/phenyl acyl tails affected receptor activation. Insights from structure-guided alanine substitution experiments led to the identification of the “quartet core” residues: L122^3.36^, F204^5.47^, W256^6.48^, and F260^6.52^ in S1P_3_, whose side chains flip in response to binding of longer acyl-tail-containing agonists, which in turn favors the activation of the receptor, and also their role in mediating subtype bias [41]. In the cysLT_1,2_ receptors (Figure 4G), the ligands also adopt a linear conformation and occupy a pocket similar to S1P, with a portion of ligand wedged between TM4 and TM5 while also engaging TM3. In the CB_1_ and CB_2_ receptors (Figure 4J), the ligands (AM11542, AM841, MDMB-Fubinaca (FUB), 9GF, WI5) are also largely linear molecules, which adopt a more bent, almost C-shaped conformation, partially wrapping around the unstructured ECL2 segment with their acyl tails extending into a similar long tunnel-like pocket observed in S1P_1_ and S1P_3_. In contrast, the LPA_1_ (Figure 4I) antagonists (ONO-3080573, ONO-97803077, ONO-9910539), CB1 antagonists (AM6538, taranabant), and PAFR (Figure 4E) antagonist (9ER) are branched with three distinct arms and adopt a Y-shaped conformation with one of the arms pointing towards the intracellular side, while the other two arms bind with a bent conformation similar to ML056 in S1P_1_. Docking studies involving CB_1_ antagonists/inverse agonists provide evidence for the roles of each of three arms viz., arm 1–high affinity, arm 2–long tunnel, and arm 3–interaction with TM1 and TM2, with potential implications for signal modulation. Further structural and functional analyses guided by molecular docking showed that the LPA_1_ orthosteric pocket could also accommodate phosphorylated CB_1_ ligands such as 2-arachidonyl phosphatidic acid (2-ALPA) leading to receptor activation, illustrating the plasticity of the ligand binding pocket [42]. In the BLT1 receptor, the ligand also adopts a linear, with one arm occupying the W^6.48^/allosteric sodium ion pocket representing a novel ligand binding mode. Wang et al. have summarized this variation by comparing structures of DP_2_, S1P_1_, LPA_1_, and CB_1_ receptors [31]. The canonical orthosteric ligand binding pocket in lipid receptors also shows significant variation in terms of both volume and residue composition. The S1P_1_-antagonist and S1P_3_-S1P structures show an amphipathic ligand binding pocket, with the phosphate and amine groups of the ligands surrounded by polar/charged amino acids, while the alkyl tail/hydrophobic moieties were packed against hydrophobic amino acids.

### 4.4. Non-Canonical or Allosteric Sites

In addition to the canonical orthosteric pocket, other pockets that are functionally and topologically distinct and can bind ligands that modulate GPCR signaling have been recognized [82]. Several such allosteric sites have been identified in all four major classes of GPCRs, and the molecules that bind to these pockets are classified based on how they influence signaling [83]. Recent advances in GPCR structural biology and pharmacology have shed light on the mechanisms of allosteric modulation in lipid GPCRs. 

Allosteric modulators are small molecules or peptides that bind to sites other than the orthosteric binding site (OBS) and exert multiple effects on GPCR signaling. Broadly, the allosteric modulators can be divided into two categories [84,85]. The first category consists of allosteric modulators that either potentiate the affinity and/or efficacy of the endogenous ligands or other orthosteric ligands and, thereby, lead to increased GPCR signaling (PAM) or exert an inverse effect (NAM). Allosteric modulators of this category lack intrinsic activity and mediate their physiological effects only when the endogenous orthosteric ligand is bound [86]. Due to this, they do not affect the spatial and temporal tone of endogenous orthosteric ligands [86] and enjoy a clear advantage over the orthosteric drugs. Moreover, their modulation is limited by the extent of cooperativity with the orthosteric ligands, leading to the ceiling effect [85], which can lead to minimal side effects. The other category [84,85] consists of direct allosteric agonists, allosteric inverse agonists, and allosteric antagonists that can independently modulate GPCR coupling to downstream signaling transduction. Another benefit of targeting allosteric sites is the lack of evolutionary pressure at these sites manifesting in poor residue conservation or greater sequence variability [87]. This presents opportunities for selective targeting of GPCR subtypes, which often have high residue conservation in OBS.

With respect to the lipid GPCRs, the structure of the human free fatty acid receptor 1 (FFA_1_; GPR40) complexed with TAK-875, an ago-allosteric modulator, shows the ligand bound to a unique non-canonical site, wedged between TMs 3, 4, and 5 and capped by ECL2 (Figure 4K). TAK-875 adopts a nearly extended conformation lying parallel to the membrane plane, with nearly 50% of the ligand remaining outside the helical bundle and periscoping towards the extracellular side of the receptor [48]. Ho et al. reported a second FFA_1_ structure complexed with a synthetic full agonist, referred to as compound 1 by the authors, bound to a second allosteric site formed by TMs 3, 4, and 5 and ICL2 [50]. In contrast to the TAK-875-binding site (A1), the second allosteric site (A2) is entirely extra-helical, lipid-facing, and located towards the intracellular side of the receptor. The authors also concluded that AM-1638, an allosteric full FFA_1_ agonist, potentially prefers the A2 site to the A1 site. Shao et al. [55] solved the crystal structure of another lipid receptor, Cannabinoid receptor 1 (CB_1_), bound to ORG27569. Interestingly, ORG27569 binds to a novel extrahelical, allosteric site at the receptor–lipid interface in the inner leaflet of the membrane. The allosteric binding site of ORG27569 lies at TM2-TM4 helix interface and partially overlaps with the cholesterol binding site in multiple GPCR structures.

Considering the importance of allosteric pockets for ligand discovery and the benefits of targeting the same, several methods have been developed for identifying such pockets. MD simulations-based probe mapping protocols [88,89,90,91] have become a valuable tool to quickly and efficiently identify and characterize allosteric binding sites in membrane proteins. These protocols extensively use cosolvent mapping, where an organic molecule is used as a probe, allowing for the identification of cavities that otherwise are inaccessible and can serve as potential binding sites for allosteric modulators. Recently, Ciancetta et al. proposed a variation of probe mapping protocols to specifically identify allosteric sites at the receptor–lipid interface, utilizing fragments of already known allosteric ligands as a probe [92]. Computational tools such as these will pave the way for fast-tracking the structure-based design of allosteric modulators.

The recent influx of crystal structures of numerous lipid and other GPCRs has resulted in a wealth of insights into their ligand recognition and binding mechanisms as well as the activation/inactivation cycle from structural, functional, and computational studies. Broadly, insights into the structural features and mechanisms employed by lipid GPCRs to capture and bind their ligands could also help in the deciphering the ligand preferences and deorphanizing certain receptors. It also could aid in understanding the ligand binding mechanisms in certain other class A receptors that are liganded by lipophilic or amphipathic molecules. For example, resolvins such as RvE1 and 2 (BLT antagonists) are known to stimulate the chemerin receptor or chemokine such as receptor 1 (CMKLR_1_) [93]. Similarly, the N-formyl peptide receptor 2 (FPR_2_) is activated by both lipoxins and resolvins, which also stimulate certain orphan receptors [94]. In addition, an in-depth understanding of lipid receptors with respect to their ligand binding pockets, both orthosteric and allosteric, and how they mediate ligand-receptor interactions and in turn receptor function, is particularly crucial for the design and development of new drug candidates and therapeutic agents [95]. 

## 5. The Challenges and Future Perspectives on the Development of GPCR-Centric Therapeutics

Over the past couple of decades, our understanding of receptor activation/inactivation, orthosteric and allosteric modulation, subtype specificity and selectivity, and biased agonism has been greatly enhanced. The sustained advances in GPCR pharmacology and structural biology, coupled with the tremendous progress in leveraging computational efforts toward understanding receptor dynamics, have provided unprecedented insights into the workings of these enigmatic cellular machines. The exponential increase in our knowledge has brought about new avenues for GPCR drug discovery and development. Traditionally, the initial phases of the drug discovery process have largely focused on the development of a wide range of small molecules showing therapeutic effects with regards to GPCRs. It is evident from the fact that the majority of FDA-approved GPCR drugs are small molecules. However, identifying small-molecule drugs with the desirable qualities of high specificity, affinity, and potency still remains the greatest challenge. As a result, there is a need to look beyond small molecules and explore novel biologics-based therapeutic approaches to target GPCRs. Herein we discuss some of the most promising approaches currently being actively explored.

### 5.1. Beyond Simple Agonism and Antagonism

One important consideration for drug discovery is biased agonism, where an agonist may selectively activate a GPCR G-protein pathway over a β-arrestin pathway, or vice versa. An example of such biased agonism or signaling is prostanoids PGD_2_ and PGE_2_, biased agonists at their cognate receptors DP and EP_2_, favoring G_α_s protein-mediated cAMP formation. Since PGD_2_ and PGE_2_ are isomers, and DP and EP_2_ are phylogenetically related, they can also cross-activate with the same biased preference [96]. This highlights another pertinent consideration of activation crosstalk among phylogenetically related GPCRs, such as the prostanoid receptors [97]. The molecular mechanism of biased agonism is an area of active research. As a further elaboration, this review [98] specifically discuses biased signaling of CB_1_ by examining its structural features and mutants.

Another consideration for drug discovery involves taking a broader look at the concept of antagonism. For example, an indirect or physiological antagonist inhibits agonist actions indirectly by blockading intermediate signaling molecules, instead of directly competing for the orthosteric site. Fingolimod, an abovementioned FDA-approved drug (S1P_1,3-5_) for multiple sclerosis, is an example of a ligand that straddles the two phenomena described above. Its phosphorylated form is a biased agonist that selectively activates G_i_-coupled inhibition of adenylyl cyclase via S1P_3_ [99]. It has also been regarded as a functional antagonist. It does not fit the definition of a classical indirect antagonist because it shares the orthosteric site with the cognate endogenous ligand S1P. Fingolimod-phosphate initially acts as agonist at S1P_1_, but it induces sustained receptor internalization, which results in desensitization of S1P, thus, in effect antagonizing it at the functional level [22].

### 5.2. Antibody-Based Therapeutics

Originating with crystal structure determination (Supplementary Tables S3 and S4), antibodies (Abs) or nanobodies (Nbs) have significantly expanded our knowledge of the molecular mechanisms of signal transduction through GPCRs. Recently, highly selective antibodies binding to extracellular GPCR epitopes (Supplementary Tables S4–S6) have emerged as a potential and attractive alternative to small-molecule therapies. Numerous strategies have been laid out to harness the improved specificity, and affinity, as well as other pharmacological properties of antibodies to target GPCRs. This has resulted in the development of multiple therapeutic GPCR-targeting Abs that are approved or are in various stages of pre-clinical trials (Supplementary Tables S5 and S6).

**Figure 5:**
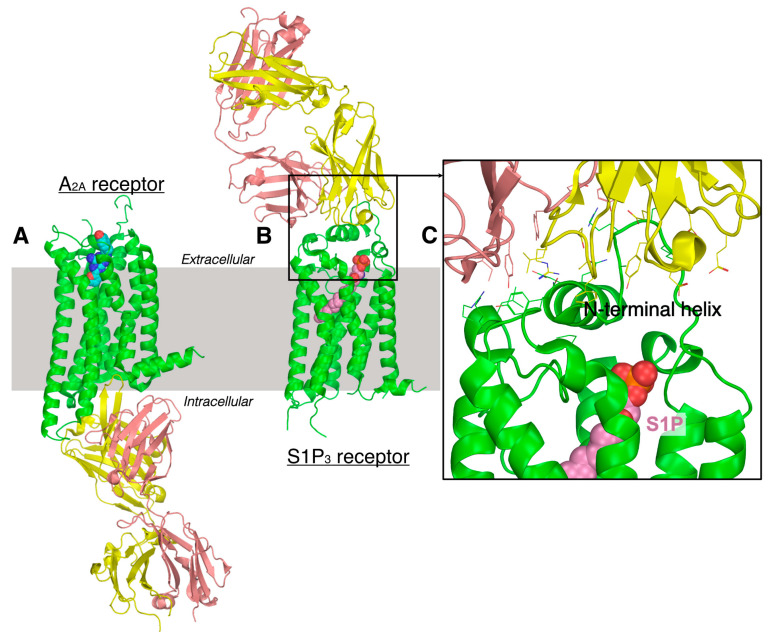
Molecular plots showing antibody binding to (**A**) intracellular and (**B**) extracellular epitope in A_2A_ adenosine (PDB ID: 3VG9) and lipid binding S1P_3_ (PDB ID: 7C4S) receptors, respectively. Receptor bound ligand molecules are shown in sphere representation. Antibody heavy and light chains are shown in yellow and brown colors. (**C**) An enlarged version of antibody receptor binding site is in lipid receptor S1PR3. The interacting side chains are shown in sticks. It is noted that the antibody does interact (within 5 Å distance) with the bound ligand (shown in pink).

The most common strategy is to alter GPCR function by modulating the binding of their ligands, thus preventing the signal-transduction-causing disease. So far, Abs/Nbs binding to extracellular epitopes have been recognized to modulate GPCR activities in multiple ways and can act as agonists, inverse agonists, or antagonists (Supplementary Table S5). Recently, Namacizumab (RYI-018), which targets and acts as an antagonist of lipid receptor CB_1_ (Supplementary Table S5), has been identified and is in preclinical trials [100,101]. However, the molecular basis of epitope recognition and molecular mechanism of GPCR modulation remained speculative due to the lack of detailed structural insights. So far, only six GPCRs (Supplementary Table S4) have been co-crystallized with antibodies bound to the extracellular side (Figure 5 and Supplementary Table S4). Out of these, only S1P_3_ belongs to lipid GPCRs (Figure 5). Interestingly, these limitedly available crystal structures have suggested that extracellular epitope in the GPCRs comprises a much smaller extracellular domain, contrary to earlier belief. This suggests that the Abs/Nbs-based therapeutics approach can be similarly extended to other GPCRs.

In another promising approach, the Fc (constant) region of the antibodies is the target of interest to develop novel therapeutics. This Fc region can recruit complement proteins and specialized Fc receptors found in innate immune cells to engage in effector functions [102,103]. These effector functions involving antibody-dependent cellular phagocytosis (ADCP), complement-dependent cytotoxicity (CDC), and antibody-dependent cell-mediated cytotoxicity (ADCC), lead to antibody-mediated killing of cells expressing a given GPCR (Supplementary Table S6). Currently, mogamulizumab is the only class-A GPCR antibody that targets CCR4 and is approved in Japan for adult T-cell leukemia-lymphoma [104] and peripheral T-cell lymphoma [105].

In a recent approach named as antibody-drug conjugate (ADC), antibodies are being employed to deliver a highly potent cytotoxic molecule (payload) attached to an antibody using a linker. The specificity of the antibody is used to bind cells that express a given GPCR, followed by internalization. The linker region is then hydrolyzed inside the lysosomes or endosomes, releasing the cytotoxic payload, leading to cell death. The development of ADCs has seen an increased interest in developing therapeutic agents for the treatment of cancer. Certain cancer cells show increased GPCR expression compared to healthy cells, thereby ADCs can be applied to specifically target them without harming healthy cells. Additionally, the conjugation of a ligand to an antibody improves the potency and specificity of the drug bound, as it increases the effective concentration of the drug at a given position. However, despite being a very promising approach, currently the ADC713 that binds CXCR4 is the only ADC that has been shown some promising results in in vitro assays [106]. This is due to the fact that ADCs suffer from multiple issues such as antigen shedding [107], which leads to an increased risk of toxicity. Further, developing antibodies that promote internalization remain a challenge due to the poorly characterized mechanism of antibody-mediated internalization.

We, therefore, summarize that antibodies have untapped potential to be used as novel therapeutics. Several studies have suggested the role of lipid-binding receptors in multiple forms of cancers. However, the therapeutic antibodies available so far have been developed to target chemokine receptors, whereas other receptors have been neglected. To bridge the gap, computational pipelines involving homology modelling, molecular docking, and molecular dynamics simulations can help expedite epitope identification and antibody design. In the future, we hope to see more receptor groups added to this list to treat cancer and other life-threatening diseases.

### 5.3. Advances in Computational Methods

Here, we provide a broad overview of recent computational works related to GPCRs from our group and others. Structural bioinformatics and other computational tools can complement biophysical and biochemical experiments to provide further insights. When available, examples involving specifically lipid GPCRs will be mentioned, but the computational techniques involving other GPCRs or even other proteins are generally applicable to lipid GPCRs as well.

When experimental structures are challenging to resolve, computational modeling may provide reasonably accurate models. Of special interest, recently, are applications of machine learning (ML) methods such as AlphaFold [108] and RoseTTAFold [109]. There are also increasing efforts to model GPCR oligomers, as reviewed by Barreto et. Al. [110]. Since GPCRs are major drug targets, molecular docking has also been routinely used to screen ligand libraries for drug discovery. Several large-library docking campaigns have been completed specifically for GPCR targets, such as CB_2_ receptor [111], MT_1_ and MT_2_ melatonin receptors [112], dopamine D_2_ and serotonin 5-HT_2A_, and κ-opioid receptors [113], the D_4_ dopamine receptor [114], and orexin receptors [115].

Molecular dynamics (MD) simulations are especially pertinent for GPCRs, which undergo large conformational changes to function. MD simulations are often reported concurrently as the structural determination, as seen in the cases of 5-HT_3A_ [116], CXCR_2_ [117], CRTH_2_/DP_2_ [32], and CB_2_ [56], where the simulations provide further conformational and binding insights. Steered MD simulations were used for investigating ligand entry and exit in CB_1_, S1P_1_, and LPA_1_ [118]. MD simulations can also be used to probe and map orthosteric and allosteric binding sites [83,91,92,119,120,121]. Advanced statistical mechanics tools such as Markov state models [122] have been used to investigate PAC1, VPAC1, and VPAC2 receptors [123], the adenosine A_2A_ receptor [124], and dopamine D_2_ and D_3_ receptors [125]. MD simulations can be used to propose molecular mechanisms of experimental observations: a recent study investigates the effect of S1P chain length on the activation of S1P receptors by functional assays and MD simulations [126] as well as another on S1P binding and the activation of S1P_1_ [127]. This review on biomolecular modeling and simulation provides a broader overview as well as more GPCR-specific examples [128].

The computational tools are also often used synergistically. Homology models can be used as target structure of molecular docking [129], such as on D_2_, 5-HT_2AR_ [130], and FFA_4_ [131]. MD simulations were used to refine the computational model for the virtual screening of the D_3_ dopamine receptor [132]. Free energy calculations were used to direct the fragment-based design of the ligands of adenosine receptors [133]. Docking was used to guide fragment evolution targeting β_1_- and β_2_-adrenergic receptors [134]. When multiple active-state structures are available, screening can occur against the multiple conformations, as in the case of the *β*_2_-adrenergic receptor [135].

In the future, we would expect to see more of this synergism and, especially, with emergent ML methods. ML scoring functions for structure-based virtual screening are in active development [136]. There are also ML ligand-based virtual screening methods such as RealVS, which is benchmarked against several GPCR targets, including, notably, lipid GPCRs S1P_3_ and CB_2_ [137]. ML applications in MD simulations include increasing the efficiency of sampling to guiding data analysis. Reviews on ML applications for virtual screening and/or MD simulations can be found here: [138,139,140].

## Figures and Tables

**Table 1:** Summary of all available experimentally determined structures of lipid GPCRs (as of September 2021).

	Cognate Receptor Name; Gene Name and Uniprot ID	Protein Engineering/Modification	Methodology	Structures
Prostanoids	Prostaglandin D2 receptor 2 (DP_2_) ^#^; PTGDR2; Q9Y5Y4	T4-lysozyme (mT4L) with 8-aa linker insert in ICL3.	X-ray crystallography (XRD)	Antagonists fevipiprant [6D26 ^Inactive^; 2.80 Å], CAY10471 [6D27 ^Inactive^; 2.74 Å] [31]
		T4-lysozyme (mT4L) with 8-aa linker insert in ICL3.	Serial femtosecond crystallography (SFX) with X-ray free electron laser (XFEL)	Agonist 15m-PGD_2_ [7M8W ^Inactive^; 2.61 A] [32]
	Prostaglandin E2 receptor EP2 subtype; PTGER2; P43116	No modifications.	Cryo-electron microscopy (cryo-EM)	Endogenous agonist PGE_2_ + G-protein G_s_ [7CX2 ^Active^; 2.80 Å], synthetic agonists taprenepag + G_s_ [7CX3 ^Active^; 2.80 Å], evatanepag (CP-533536) + G_s_ [7CX4 ^Active^; 2.90 Å] [33]
	Prostaglandin E2 receptor EP3 subtype; PTGER3; P43115	Thermostabilized apocytochrome b_562_RIL (bRIL) insert in ICL3; N- and C-terminal truncation; four thermostabilizing mutations	Lipidic cubic phase crystallization (LCP); XRD	Agonist PGE_2_ [6AK3 ^Active^; 2.90 Å] [34]
		T4-lysozyme insertion in ICL3; C-terminal truncation.	LCP; XFEL	Agonist misoprostol [6M9T ^Active^; 2.5 Å] [35]
	Prostaglandin E2 receptor EP4 subtype; PTGER4; P35408	Stabilizing anti-human EP4 antibody (IgG#001); removal of *N*-glycosylation site; ICL3, N- and C-terminal truncation; two thermostabilizing point mutations	LCP; XRD	Antagonists ONO-9990614 [5YHL ^Inactive^; 4.20 Å], ONO-AE3-208 [5YWY ^Inactive^; 3.20 Å] [36];
		G_s_-stabilizing nanobody Nb35	Cryo-EM	Agonist PGE_2_ + G_s_ + nanobody Nb35 [7D7M ^Active^; 3.30 Å] [37]
	Thromboxane A2 receptor; TBXA2R; P21731	Thermostabilized b_562_RIL (bRIL) insert in N-terminal [6IIV]; rubredoxin insert in ICL3 [6IIV]; C-terminus truncation; one thermostabilizing point mutation	LCP; XRD	Antagonists ramatroban [6IIU ^Intermediate^; 2.50 Å] dalotroban [6IIV ^Intermediate^, 3.00 Å] [38]
Platelet-activating factor	Platelet-activating factor receptor; PTAFR; P25105	Flavodoxin insert [5ZKP] and T4-lysozyme [5ZKQ] insert in ICL3;	LCP; XRD	Antagonists SR 27417 [5ZKP ^Other^; 2.81 Å] and BT-491 [5ZKQ ^Intermediate^; 2.90 Å] [39]
Lysophospholipids	Sphingosine 1-phosphate receptor 1; S1PR1; P21453	T4-lysozyme insert in ICL3	XRD [3V2W]; microdiffraction [3V2Y]	Antagonist ML056 [3V2W ^Inactive^, 3.35 Å; 3V2Y ^Inactive^, 2.80 Å] [40]
	Sphingosine 1-phosphate receptor 3; S1PR3; Q99500	C-terminus truncation; removal of *N*-glycosylation site; stabilizing Fab antibody fragment (Fab AS55)	LCP; XRD	Natural agonist d18:1 S1P + Fab AS55 [7C4S ^Active^; 3.2 Å] [41]
	Lysophosphatidic acid receptor 1; LPAR1; Q92633	bRIL insert in ICL3; C-terminus truncation; engineered disulfide bridges [4Z36]; stabilizing antagonists	XRD	Selective antagonists ONO-9780307 [4Z34 ^Inactive^; 3.0 Å], ONO-9910539 [4Z35 ^Inactive^; 2.90 Å], ONO-3080573 [4Z36 ^Inactive^; 2.90 Å] [42]
	Lysophosphatidic acid receptor 6; *dr*lpar6a; Q08BG4	T4-lysozyme insert in ICL3	LCP; XRD	Apo state [5XSZ ^Intermediate^; 3.20 Å] [43]
Leukotrienes	Leukotriene B4 receptor 1; *cp*LTB4R; Q9WTK1	T4-lysozyme insert in ICL3; N-terminus truncation; thermostabilizing point mutations	LCP; XRD	Antagonist BIIL260 [5X33 ^Inactive^; 3.70 Å] [44]
	Leukotriene B4 receptor 1; LTB4R; Q15722	Flavodoxin insert in ICL3; N- and C-termini truncation; thermostabilizing point mutations	LCP; XRD	Antagonist ML-D-046 [7K15 ^Inactive^; 2.88 Å] [45]
	Cysteinyl leukotriene receptor 1; CYSLTR1; Q9Y271	Thermostabilized b_562_RIL (bRIL) insert in ICL3; C-terminal truncation.	LCP; SFX with XFEL	Antagonists pranlukast [6RZ4 ^Intermediate^; 2.70 Å] and zafirlukast [6RZ5 ^Intermediate^; 2.53 Å] [46]
	Cysteinyl leukotriene receptor 2; CYSLTR2; Q9NS75	Thermostabilized b_562_RIL (bRIL) insert in ICL3; stabilizing mutations; N- and C-termini truncation	LCP; XRD	Antagonists ONO-2570366 [6RZ6 ^Intermediate^, 6RZ7 ^Intermediate^; 2.43 Å], ONO-2080365 [6RZ8 ^Intermediate^; 2.70 Å], and ONO-2770372 [6RZ9 ^Intermediate^; 2.73 Å] [47]
Free fatty acids	Free fatty acid receptor 1 (GRP40); O14842; FFAR1	T4-lysozyme insert in ICL3	LCP; XRD	Allosteric partial agonist TAK-875 [4PHU ^Intermediate^; 2.30 Å] [48]
		T4-lysozyme insert in ICL3; three thermostabilizing mutations;	LCP; XRD	AgoPAM AP8 and partial agonist MK-8666 [5TZY ^Inactive^; 3.22 Å], MK-8666 [5TZR ^Intermediate^; 2.20 Å] [49];
		T4-lysozyme insert in ICL3	LCP; XRD	Full agonist “compound 1” [5KW2 ^Intermediate^; 2.76 Å] [50]
Cannabinoids	Cannabinoid receptor 1 (CB_1_); CNR1; P21554	Flavodoxin insert in ICL3; stabilizing antagonist; N- and C-termini truncation; four thermostabilizing mutations	LCP; XRD	Antagonist AM6538 [5TGZ ^Inactive^; 2.80 Å] [51]
		Thermostable *P. abyssi* glycogen synthase (PGS) domain insert in ICL3; N- and C-termini truncation; one thermostabilizing mutation	LCP; XRD	Inverse agonist taranabant [5U09 ^Inactive^; 2.6 Å] [52]
		Flavodoxin insert in ICL3; N- and C-termini truncation; four thermostabilizing mutations; stabilizing agonists	LCP; XRD	Agonists AM11542 [5XRA ^Active^; 2.80 Å], AM841 [5XR8 ^Active^; 2.95 Å] [53]
		Stabilizing single-chain variable fragment scFv16	Single-particle cryo-EM	MDMB-Fubinaca (FUB) + G_i_ + scFv16 [6N4B ^Active^; 3.0 Å] [54];
		Five stabilizing mutations	LCP; XRD	NAM ORG27569 [6KQI ^Inactive^; 3.245 Å] [55];
		BRIL insert in N-terminus; ; CB_1_-Gi stabilized by svFv16	Single-particle cryo-EM	Agonist AM841 + G_i_ + svFc16 [6KPG ^Active^; 3.00 Å] [56]
	Cannabinoid receptor 2 (CB2); CNR2; P34972	Rationally designed stabilizing antagonist; T4-lysozyme insert in ICL3	LCP; XRD	Antagonist AM10257 [5ZTY ^Inactive^; 2.80 Å] [57];
		CB_2_-Gi stabilized by svFv16	Cryo-EM	Agonist WIN 55,212-2 + Gi + svFv16 [6PT0 ^Active^, 3.2 Å] [58]
		BRIL insert in N-terminus; CB_2_-Gi stabilized by svFv16	X-ray [6KPC]; Single-particle cryo-EM	Agonist AM12033 [6KPC ^Active^; 3.20 Å], Agonist AM12033 + G_i_ + svFc16 [6KPF ^Inactive^; 2.90 Å] [56]

^#^ short-form of receptor names are provided in parentheses. ^Active^ Indicates that solved structure is in its active conformation. ^Inactive^ Indicates that solved structure is in its inactive conformation. ^Intermediate^ Indicates that solved structure is in an intermediate conformation.

## Data Availability

Data sharing not applicable.

## Supplementary Materials

The following are available online at https://www.mdpi.com/article/10.3390/ph15010012/s1, Table S1: Updated list of approved lipid GPCR drugs as of September 2021, Table S2: Summary of disulfide bridges in lipid GPCR structures, Table S3: GPCR crystal structures co-crystallized with antibody/nanobody bound to intracellular epitopes, Table S4: GPCR crystal structures co-crystallized with antibodies bound to extracellular epitopes, Table S5: Therapeutic antibodies that modulate class A GPCR functions. Table S6: Class A GPCR antibodies that harness effector functions to achieve therapeutic effects [36,41,141,142,143,144,145,146,147,148,149,150,151,152,153,154,155,156,157,158,159,160,161].
