# Supplementary material for: Recent Advances in Structure, Function, and Pharmacology of Class A Lipid GPCRs: Opportunities and Challenges for Drug Discovery
Source: Pharmaceuticals (Basel). 2021 Dec 22;15(1):12. doi: 10.3390/ph15010012 (PMC8779880; doi:10.3390/ph15010012)
Supplement: Supplementary file 1 [file pharmaceuticals-15-00012-s001.zip › pharmaceuticals-1510871 suppl (1).pdf]

Recent Advances in Structure, Function, and Pharmacology of Class A lipid GPCRs: Opportunities and Challenges for Drug Discovery

R. N. V. Krishna Deepak [1,*,†], Ravi Kumar Verma [1,†], Yossa Dwi Hartono [1,2,3,†], Wen Shan Yew [2,3], Hao Fan [1,2,*]

1 Bioinformatics Institute, A*STAR, 30 Biopolis Street, Matrix #07-01, Singapore 138671, Singapore;
ravikumarv@bii.a-star.edu.sg (R.K.V.); yossadh@bii.a-star.edu.sg (Y.D.H.)
2 Synthetic Biology Translational Research Programme, Yong Loo Lin School of Medicine,
National University of Singapore, 14 Medical Drive, Singapore 117599, Singapore; wenshanyew@nus.edu.sg
3 Department of Biochemistry, Yong Loo Lin School of Medicine, National University of Singapore, 8 Medical Drive, Singapore 117597, Singapore
* Correspondence: krishnad@bii.a-star.edu.sg (R.N.V.K.D.); fanh@bii.a-star.edu.sg (H.F.)
† These authors contribute equally to this work.

Supplementary Table S1: Updated list of approved lipid GPCR drugs as of September 2021.

GPCR target	Drug	Disease/agent (DRUGBANK)	Status
CB_{1-2}<br>GPR18<br>GPR55	Nabilone/dronabinol/THC	anorexia, antinausea	FDA 1985 (DRUGBANK)
CB_1	Tolazoline	vasodilator	FDA 1948 (IUPHAR)
CB_1<br>GPR55	Rimonabant	anti-obesity	Not approved in US but approved in 38 countries
CB_{1-2}	Cannabidiol	epilepsy, analgesic	FDA 2018 (DRUGBANK)
CB_2	Lasofoxifene	osteoprosis, vaginal atrophy	EMA 2009 (DRUGBANK)
$CysLT_{1-2}$	Zafirlukast	prophylaxis, asthma	FDA 1996 (FDA website)
$CysLT_1$	Fosphenytoin	epilepsy	FDA 2001 (FDA website)
$CysLT_1$	Montelukast	asthma, allergic rhinitis	FDA 1998 (DRUGBANK)
$CysLT_{1-2}$	Pranlukast	asthma, allergic rhinitis	Not approved in US but approved in Japan
$CysLT_1$	Cinalukast	asthma	Not approved in US
$CysLT_{1-2}$<br>DP_1	Nedocromil	allergic conjunctivitis	FDA 1997 (FDA website)
FFA_1	Rosiglitazone	maintain glycemic control in type 2 diabetes	FDA 1999 (FDA website)
FFA_1	Icosapent	nutraceutical	FDA 2012 (FDA website)
GPR18	Glycine	nutraceutical	
GPR35	Bumetanide	edema	FDA 2002 (FDA website)
GPR35	Furosemide	diuretic, hypertension and edema	FDA 1999 (FDA website)
GPR35	Pyrantel	anthelmintic	FDA approved for vet
GPR68	Lorazepam	panic disorders, severe anxiety, and seizures	FDA 1977 (FDA website)
PAF	Rupatadine	allergic rhinitis	PMDA 2017 (KEGG)

DP_{1-2}<br>EP_{1-4}<br>FP<br>TP	Dinoprostone (PGE_2)	induce labor or abortion, nonmetastatic gestational trophoblastic disease	FDA 1977 (FDA website)
DP_{1-2}<br>$EP_{1-2,4}$<br>IP	Alprostadil (PGE_1)	erectile dysfunction	FDA 2002 (FDA website)
DP_{1-2}<br>EP_{1-4}<br>FP<br>IP<br>TP	Iloprost	pulmonary arterial hypertension	FDA 2004 (FDA website)
DP_1<br>EP_{1-4}<br>IP	Treprostinil	pulmonary arterial hypertension	FDA 2002 (FDA website)
DP_2	Indomethacin	anti-inflammatory	FDA 2010 (FDA website)
DP_2<br>TP	Ramatroban	anti-allergic	PMDA 2000 (KEGG)
DP_2	Sulindac	osteoarthritis, rheumatoid arthritis, ankylosing spondylitis, acute subacromial bursitis or supraspinatus tendinitis, and acute gouty arthritis	
$EP_{1,4}$<br>IP	Epoprostenol (PGI_2)	vasodilator, platelet aggregation inhibitor	discontinued (FDA website)
EP_1	Bupivacaine	local anesthetic	FDA 1983 (FDA website)
EP_1	Carboprost tromethamine	postpartum uterine hemorrhage	FDA 1979 (FDA website)
$EP_{1,3}$<br>FP	Bimatoprost	hypotrichosis of the eyelashes and intraocular pressure in open angle glaucoma or ocular hypertension	FDA 2001 (FDA website)
EP_{1-4}	Limaprost	thromboangiitis obliterans, acquired lumbar spinal canal stenosis, and other ischemic conditions	discontinued (DRUGBANK)
EP_{2-4}	Misoprostol (methyl ester)	reduce the risk of NSAID-induced gastric ulcers	FDA 1988 (FDA website)

$EP_{2,3}$	Gemeprost	cervical dilation and, in combination with mifepristone, for pregnancy termination	Not approved in US but approved in Japan (DRUGBANK)
EP_2	Omidenepag isopropyl	glaucoma	PMDA 2018 (KEGG)
EP_4	Grapiprant	osteoarthritis	FDA 2016 for vet (DRUGBANK)
FP	Carboprost	postpartum uterine hemorrhage	FDA 1979 (FDA website)
FP	Isoflurane	general anesthetic	FDA 1979 (FDA website)
FP	Trimipramine	antidepressant	FDA 1979 (FDA website)
FP	Metoclopramide	gastroesophageal reflux disease, prevention of nausea and vomiting, and to stimulate gastric emptying	FDA 1980 (DRUGBANK)
FP	Tafluprost	ocular hypertension or open-angle glaucoma	FDA 2012 (DRUGBANK)
FP	Latanoprost	ocular hypertension	FDA 1998 (DRUGBANK)
FP	Travoprost	ocular hypertension or open-angle glaucoma	FDA 2006 (FDA website)
FP<br>IP	Dinoprost tromethamine	induce a second trimester abortion	discontinued (FDA website)
IP	Insulin lispro	control hyperglycemia in diabetes mellitus	FDA 2015 (FDA website)
IP	Olanzapine	schizophrenia, bipolar 1 disorder	FDA 1996 (DRUGBANK)
IP	Remifentanil	analgesic	FDA 1996 (FDA website)
IP	Selexipag	pulmonary arterial hypertension	FDA 2015 (DRUGBANK)
$S1P_{1,5}$	Ozanimod hydrochloride	Multiple Sclerosis (MS) and inflammatory bowel disease	FDA 2020 (DRUGBANK)
$S1P_{1,3\text{-}5}$	Fingolimod	multiple sclerosis	Phase 2 clinical trials (DRUGBANK)
$S1P_1$	Asfotase alfa	perinatal/infantile and juvenile onset hypophosphatasia	FDA 2015 (DRUGBANK)
$S1P_{1,5}$	Siponimod fumarate	multiple sclerosis	FDA 2019 (DRUGBANK)
$S1P_1$	Ponesimod	multiple sclerosis	FDA 2021 (DRUGBANK)
TP	Ridogrel	prevention of systemic thrombo-embolism and as an adjunctive agent to thrombolytic therapy in acute myocardial infarction	Phase III discontinued in 2002
TP	Seratrodast	asthma	Not approved in US

Supplementary Table S2: Summary of disulfide bridges in lipid GPCR structures.

NAME	DISULFIDE BRIDGE						
	NT-ECL1	NT-ECL2	NT-TM5	TM1-TM7	TM3-ECL2	ECL2	ECL3
$S1P_1$	-	-	-	-	-	C184-C191	C282-C287
$S1P_3$	-	-	-	-	-	C178-C185	C269-C274
LPA_1	-	C24-C190	-	-	-	C188-C195	C284-C287
LPA_6	-	-	-	-	C99-C178	-	-
FFA_1	-	-	-	-	C79-C170	-	-
PAFR	-	-	-	-	C90-C173	-	-
DP_2	-	-	C11-C199	-	C104-C182	-	-
EP_2	-	-	-	-	C109-C187	-	-
EP_3	-	-	-	-	C130-C208	-	-
EP_4	-	-	-	-	C92-C170	-	-
TP	C11-C102	-	-	-	C105-C183	-	-
BLT_1	-	-	-	-	C92-C170	-	-
$CysLT_1$	-	-	-	C14-C267	C96-C173	-	-
$CysLT_2$	-	-	-	C31-C279	C111-C187	-	-
CB_1	-	-	-	-	-	C257-C264	-
CB_2	-	-	-	-	-	C174-C179	-

Supplementary Table S3: GPCR crystal structures co-crystallized with antibody/nanobody bound to intracellular epitopes.

	Receptor	Receptor family	PDB	Resolution	State	Antibodies
**1**	A_{2A}	Adenosine	3VG9 [141]	2.7	Inactive	Antibody
**2**	M_2	Acetylcholine (muscarinic)	4MQT [142]	3.7	Active	Nanobody
			4MQS [142]	3.5	Active	Nanobody
**3**	β_1	Adrenoceptors	7BU6 [143]	2.7	Active	Nanobody
			7BU7 [143]	2.6	Active	Nanobody
			7BTS [143]	3.1	Active	Nanobody
**4**	β_2	Adrenoceptors	5JQH [144]	3.2	Inactive	Nanobody
			3KJ6 [145]	3.4	Inactive	Antibody
			2R4R [146]	3.4	Inactive	Antibody
			2R4S [146]	3.4	Inactive	Antibody
			6N48 [147]	3.2	Active	Nanobody
			4QKX [148]	3.3	Active	Nanobody
			4LDE [149]	2.8	Active	Nanobody
			4LDL [149]	3.1	Active	Nanobody
			4LDO [149]	3.2	Active	Nanobody
			3P0G [150]	3.5	Active	Nanobody
			6MXT [151]	3.0	Active	Nanobody
**5**	AT_1	Angiotensin	6OS0 [152]	2.9	Active	Nanobody
			6DO1 [152]	2.9	Active	Nanobody
			6OS1 [153]	2.8	Active	Nanobody
			6OS2 [153]	2.7	Active	Nanobody
**6**	Ghrelin	Ghrelin	6KO5 [154]	3.3	Inactive	Antibody
**7**	k-opioid (KOR1)	Opioid	6B73 [155]	3.1	Active	Nanobody
			6VI4 [156]	3.3	Inactive	Antibody

Supplementary Table S4: GPCR crystal structures co-crystallized with antibodies bound to extracellular epitopes.

	Receptor	Receptor family	PDB	Resolution	State	Antibodies
**1**	5-HT_{2B}	5-Hydroxytryptamine	5TUD [157]	3.0	Active	Antibody
**2**	AT_2	Angiotensin	5XJM [158]	3.2	Active	Antibody
			6JOD [159]	3.2	Active	Antibody
**3**	D_2	Dopamine	7DFP [160]	3.1	Inactive	Antibody
**4**	PAR_2 (GPR11)	Proteinase-activated	5NJ6 [161]	4.0	Intermediate	Antibody
**5**	EP_4	Prostanoid	5YWY [36]	3.2	Inactive	Antibody
			5YHL [36]	4.2	Inactive	Antibody
**6**	$S1P_3$	Lysophospholipid (S1P)	7C4S [41]	3.2	Active	Antibody

Supplementary Table S5: Therapeutic antibodies that modulate class A GPCR functions.

Drug	Type of molecule	Mode of action	Indication	Target Receptor	Receptor class	Phase
ALX-0651	Nb	Antagonist	Antineoplastic	CXCR4	Chemokine	Phase I (terminated)
Avdoralimab	mAb	Antagonist	Antineoplastic	C5A1	Complement peptide	Phase I (recruiting)
BNC101, ET-101	mAb	Antagonist	Antineoplastic	LGR5 (GPR49, GPR67)	Class A orphans	Phase I (terminated)
Mogamulizumab	mAb	Antagonist	Antineoplastic	CCR4	Chemokine	Approved
MEDI3185	Ab	Antagonist	Antineoplastic	CXCR4	Chemokine	Preclinical
Namacizumab, RYI-018, Nimacimab, JNJ2463	mAb	Antagonist	Non-alcoholic fatty liver disease; Non-alcoholic steatohepatitis	CB_1	Cannabinoid	Preclinical
Plozalizumab, MLN1202	mAb	Antagonist	Antineoplastic	CCR2	Chemokine	Phase II (completed)
Leronlimab	mAb	Antagonist	HIV infections	CCR5	Chemokine	Phase III (recruiting)
Ulocuplumab	mAb	Antagonist	Antineoplastic	CXCR4	Chemokine	Phase I (ongoing)

Nb = nanobody
mAb = monoclonal antibody

Supplementary Table S6: Class A GPCR antibodies that harness effector functions to achieve therapeutic effect.

Inhibitor	Type of molecule	Mode of action	Indication	Target Receptor	Receptor class	Phase
Ulocuplumab	mAb	ADCC	Antineoplastic	CXCR4	Chemokine	Phase I (ongoing)
Mogamulizumab	mAb	ADCC	Antineoplastic	CCR4	Chemokine	Approved
LY2624587	Ab	Apoptosis	Antineoplastic	CXCR4	Chemokine	Phase I (completed)
hz-515H7, F50067	mAb	ADCC and CDC	Antineoplastic	CXCR4	Chemokine	Preclinical
X7Ab	Ab	ADCC, CDC, and ADCP	Antineoplastic	ACKR3 (CXCR7, GP159)	Chemokine	Preclinical

Nb = nanobody
mAb = monoclonal antibody
